# Differential analysis of lung adenocarcinoma and lung squamous cell carcinoma based on serum tumor markers: diagnostic performance and clinical utility

**DOI:** 10.1016/j.clinsp.2026.101078

**Published:** 2026-09-02

**Authors:** Zhuoying Chen, Li Chen, Lichun Wu, Ke Zhang

**Affiliations:** a Department of Blood Transfusion, Sichuan Clinical Research Center for Cancer, Sichuan Cancer Hospital & Institute, Sichuan Cancer Center, University of Electronic Science and Technology of China, Chengdu, China; b Department of Laboratory Medicine, Sichuan Clinical Research Center for Cancer, Sichuan Cancer Hospital & Institute, Sichuan Cancer Center, University of Electronic Science and Technology of China, Chengdu, China

**Keywords:** Non-small cell lung cancer, Serum tumor markers, ROC curve, Decision curve analysis, Cost-effectiveness

## Abstract

**Objective:**

This study evaluated the performance of Serum Tumor Markers (STMs) in differentiating Lung Adenocarcinoma (LUAD) from Lung Squamous Cell Carcinoma (LUSC), as well as their cost-effectiveness and net clinical benefit.

**Methods:**

The authors retrospectively analyzed 1009 patients with pathologically confirmed Non-Small Cell Lung Cancer (NSCLC), including 796 cases of LUAD and 213 cases of LUSC.

**Results:**

Serum levels of Cytokeratin 19 Fragment (CYFRA21–1), Squamous Cell Carcinoma Antigen (SCCA), Neuron-Specific Enolase (NSE), and Pro-Gastrin-Releasing Peptide (ProGRP) were significantly higher in LUSC, whereas Carcinoembryonic Antigen (CEA) was higher in LUAD (all *p* < 0.05). Among individual markers, SCCA and CYFRA21–1 showed the best discrimination, with Areas Under the Receiver Operating Characteristic Curve (AUCs) of 0.77 and 0.76, respectively, while CEA showed limited performance (AUC = 0.55). A multivariate model incorporating age, CEA, CYFRA21–1, and SCCA achieved an AUC of 0.82 (95% Confidence Interval [95% CI]: 0.79–0.85). At the selected threshold, sensitivity and specificity were 0.76 and 0.72, respectively. Model calibration was acceptable overall despite a statistically significant Hosmer-Lemeshow test (*p* = 0.002). Decision Curve Analysis (DCA) demonstrated a higher net benefit for the model than strategies of performing immunohistochemistry in all patients or none across threshold probabilities of 0.1–0.7. A stepwise diagnostic strategy ‒ initial STM testing followed by immunohistochemistry ‒ was associated with reduced projected diagnostic costs.

**Conclusion:**

CYFRA21–1 and SCCA were the most informative STMs for distinguishing LUAD from LUSC. A parsimonious multivariate model demonstrated good discrimination, acceptable calibration, and clinical net benefit. External validation and prospective evaluation are warranted before clinical implementation.

## Highlights

• Serum tumor markers differentiate lung adenocarcinoma from lung squamous cell carcinoma.
• STM panel outperforms single markers in subtype prediction.
• STM-based workflow enables non-invasive, cost-effective NSCLC subtype identification.

## Introduction

Lung cancer remains a leading cause of cancer-related morbidity and mortality worldwide. NSCLC accounts for approximately 80%–85% of all lung cancer cases.^1^ Among NSCLC, LUAD and LUSC are the two predominant histological subtypes, which differ substantially in terms of pathogenesis, molecular alterations, therapeutic strategies, and prognosis.^2^

LUAD is more frequently observed in women and never-smokers and is commonly associated with actionable driver mutations, such as EGFR and ALK, enabling the use of targeted therapies and immunotherapy.^2^^,^^3^ In contrast, LUSC predominantly affects smokers, harbors fewer targetable genomic alterations, and often relies more heavily on immunotherapy and conventional chemotherapy.^4^^,^^5^ Given these clinically meaningful differences, accurate histological subtyping is essential for individualized treatment selection and prognostic evaluation.

Histopathological examination remains the gold standard for NSCLC subtyping. However, in routine clinical practice, definitive classification may be challenging in certain scenarios, such as when biopsy specimens are limited, tumor tissue is poorly preserved, or morphological features are ambiguous.^6^ These limitations are particularly relevant in patients undergoing minimally invasive procedures or those with advanced disease, highlighting the need for complementary diagnostic approaches.

With the advancement of precision medicine, STMs have attracted increasing attention as auxiliary tools for lung cancer diagnosis and subtyping. STMs are minimally invasive, cost-effective, easy to obtain, and highly reproducible across clinical settings, making them attractive candidates for large-scale clinical application.^7^^,^^8^ Several commonly used STMs ‒ including CEA, CYFRA21–1, SCCA, NSE, and ProGRP ‒ have demonstrated differential expression patterns across NSCLC subtypes, suggesting potential value in histological discrimination and clinical decision-making.^7^^,^^9^^,^^10^

Nevertheless, the clinical utility of individual STMs remains limited. Most single biomarkers show suboptimal sensitivity or specificity and are insufficient for independent use in routine NSCLC subtyping. As a result, reliance on a single STM rarely provides robust diagnostic confidence, restricting its standalone clinical applicability.

To address these limitations, increasing attention has been directed toward biomarker signatures that integrate multiple STMs using statistical modeling or machine learning approaches. Such multivariable strategies may improve diagnostic discrimination, enable risk stratification, and support clinical decision-making by leveraging complementary information from multiple biomarkers.^11^^,^^12^

In this study, the authors retrospectively analyzed the medical records of 1009 patients with pathologically confirmed NSCLC. The aims of this study were: 1) To evaluate and compare the diagnostic performance of five commonly used STMs (CEA, CYFRA21–1, SCCA, NSE, and ProGRP) in distinguishing LUAD from LUSC; 2) To develop and validate a parsimonious multivariable diagnostic model for histological subtyping based on clinical and serum biomarker data; and 3) To assess the clinical utility and cost feasibility of a stepwise diagnostic strategy incorporating STM testing and immunohistochemistry.

## Methods

### Study design and cohort

This single-center retrospective study included 1009 consecutive patients with NSCLC diagnosed at Sichuan Cancer Hospital between January 2024 and December 2024. The study was designed to evaluate the diagnostic value of baseline serum tumor markers for histological subtyping at initial presentation.

Inclusion criteria were: 1) Histologically confirmed LUAD or LUSC; 2) Newly diagnosed patients at first admission; 3) Availability of a complete baseline STM panel obtained prior to any anti-tumor treatment; and 4) Complete clinicopathological records.

Exclusion criteria included: 1) Prior chemotherapy, radiotherapy, targeted therapy, immunotherapy, or surgery before serum sampling; 2) History of other active malignancies; 3) Benign or malignant conditions known to significantly influence STM levels (e.g., hepatobiliary disease, chronic inflammatory lung disease, severe renal dysfunction, or neuroendocrine tumors); and 4) Incomplete clinical data.

This study was approved by the Medical Research and New Technology Ethics Committee of Sichuan Cancer Hospital (Approval n° SCCHEC-02–2024–130). Informed consent was waived owing to the retrospective design and anonymized data use.

### Variables and assays

Baseline demographic and clinical data were extracted from electronic medical records. Peripheral venous blood samples were collected in the morning after overnight fasting, allowed to clot, and centrifuged within 2 hours. Serum samples were analyzed immediately or stored at 2–8 °C and analyzed within 24 hours.

Serum concentrations of CEA, CYFRA21–1, SCCA, NSE, and ProGRP were measured using chemiluminescence immunoassays on the Mindray CL-8000i analyzer (Mindray Bio-Medical Electronics Co., Ltd., Shenzhen, Guangdong, China). All assays were performed following standard operating procedures and internal quality control protocols.

### Pathological diagnosis

All diagnoses were based on histological examination of biopsy or surgical specimens processed using standard formalin fixation and paraffin embedding. Hematoxylin and eosin staining was performed routinely. When morphology was insufficient, immunohistochemical staining (TTF-1, napsin A, p40, CK5/6) was used. Diagnoses followed the 2021 WHO classification and were independently reviewed by two pathologists.

### Statistical analysis

Continuous variables were first assessed for normality using the Shapiro-Wilk test. Normally distributed variables are presented as mean ± standard deviation and were compared using the independent-samples *t*-test. Non-normally distributed variables are presented as median (interquartile range) and were compared using the Mann-Whitney *U test*. Categorical variables are expressed as counts and percentages and were compared using Pearson’s Chi-Square test.

The diagnostic performance of individual serum tumor markers was evaluated using the Receiver Operating Characteristic (ROC) curve analysis. Areas Under the ROC Curve (AUCs) and 95% Confidence Intervals were calculated using the DeLong method. Optimal cut-off values were determined based on the Youden index.

Binary logistic regression analysis was performed to identify variables associated with lung squamous cell carcinoma. Variables significant in univariable analysis were entered into a multivariable model. Model selection was based on the Akaike information criterion to balance goodness-of-fit and parsimony. Results are presented as odds ratios with 95% Confidence Intervals.

Model discrimination was assessed using AUC, and calibration was evaluated using calibration curves and the Hosmer-Lemeshow goodness-of-fit test. Clinical utility was assessed using decision curve analysis across a range of threshold probabilities. All analyses were conducted using R software (version 4.5). This study was conducted in accordance with the STARD (Standards for Reporting Diagnostic Accuracy Studies) guidelines.

## Results

### Baseline characteristics

**Fig. 1:**
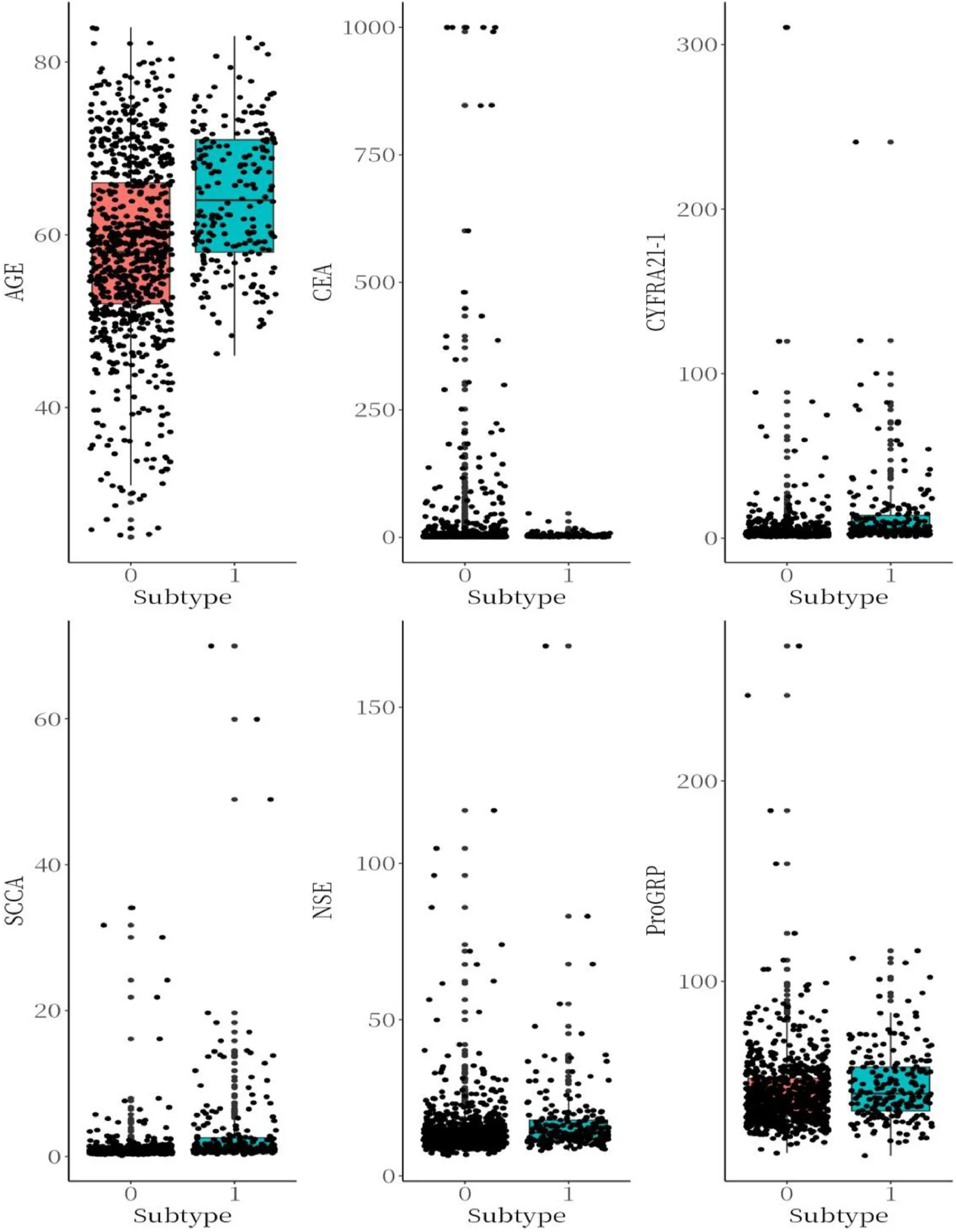
Distribution of clinical variables and serum tumor marker levels in the studied cohort. Boxplots show the distribution of age, CEA, CYFRA21–1, SCCA, NSE and ProGRP in patients with LUAD (0) and LUSC (1). Boxes represent the Interquartile Range (IQR); horizontal lines denote the median; and whiskers extend to the 1.5 × IQR; points indicate outliers. LUSC, Lung Squamous Cell Carcinoma; LUAD, Lung Adenocarcinoma.

Baseline characteristics are shown in Table 1. A total of 1009 patients (796 patients with LUAD and 213 with LUSC) were included in the analysis. Patients with LUSC were significantly older than patients with LUAD (64.3 ± 7.99 vs. 58.1 ± 10.80 years, *p* < 0.001). The levels of CYFRA21–1, SCCA, NSE and ProGRP were significantly higher in the LUSC group than in the LUAD group (*p* < 0.001 or *p* = 0.004), while CEA was higher in patients with LUAD (*p* = 0.016). The proportion of men was significantly higher in the LUSC group (91.55%vs. 43.47%, *p* < 0.001). The distribution of each variable in the two groups is shown in Fig. 1.

**Table 1:** Baseline demographic and clinical characteristics of patients with LUAD and LUSC.

Variables	Total (n = 1009)	0 (n = 796)	1 (n = 213)	Statistic	p
Age, Mean ± SD	59.40 ± 10.58	58.09 ± 10.80	64.30 ± 7.99	*t* = −9.28	<0.001
CEA, M (Q₁, Q₃)	2.21 (1.30, 4.17)	2.03 (1.20, 4.48)	2.62 (1.77, 3.74)	*Z* = −2.40	0.016
CYFRA21–1, M (Q₁, Q₃)	2.68 (1.86, 4.85)	2.41 (1.74, 3.72)	5.31 (3.01, 13.74)	*Z* = −11.86	<0.001
SCCA, M (Q₁, Q₃)	0.75 (0.56, 1.07)	0.70 (0.53, 0.92)	1.22 (0.79, 2.57)	*Z* = −11.99	<0.001
NSE, M (Q₁, Q₃)	12.89 (10.94, 16.04)	12.71 (10.76, 15.66)	13.84 (11.66, 17.85)	*Z* = −4.20	<0.001
ProGRP, M (Q₁, Q₃)	41.56 (33.22, 52.65)	41.04 (32.72, 51.53)	43.89 (35.25, 57.12)	*Z* = −2.90	0.004
Sex, n (%)				χ^2^ = 156.21	<0.001
0	468 (46.38)	450 (56.53)	18 (8.45)		
1	541 (53.62)	346 (43.47)	195 (91.55)		

t, *t*-test; *Z*, Mann-Whitney test; χ^2^, Chi-Square test; SD, Standard Deviation; M, Median; Q₁, 1st Quartile, Q₃, 3st Quartile.

Clinical variables were collected at first admission prior to treatment. All patients were newly diagnosed cases.

### Individual STMs

**Fig. 2:**
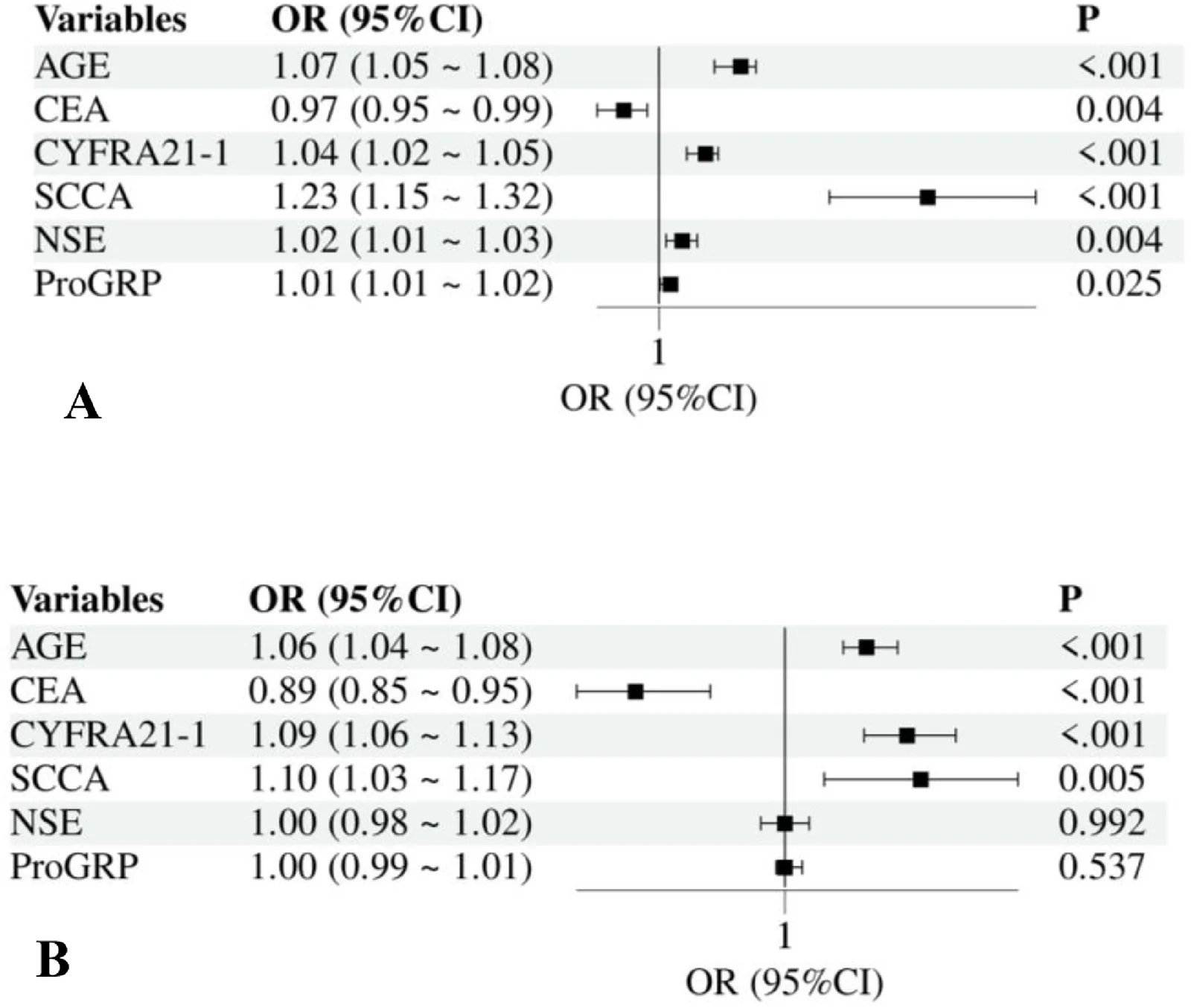
Forest plots of logistic regression odds ratios for LUSC versus LUAD. Visualizations of univariate and multivariate Logistic regression results are presented in forest plots (A–B), which clearly show the direction of effect and 95% Confidence Intervals for each variable.

**Fig. 3:**
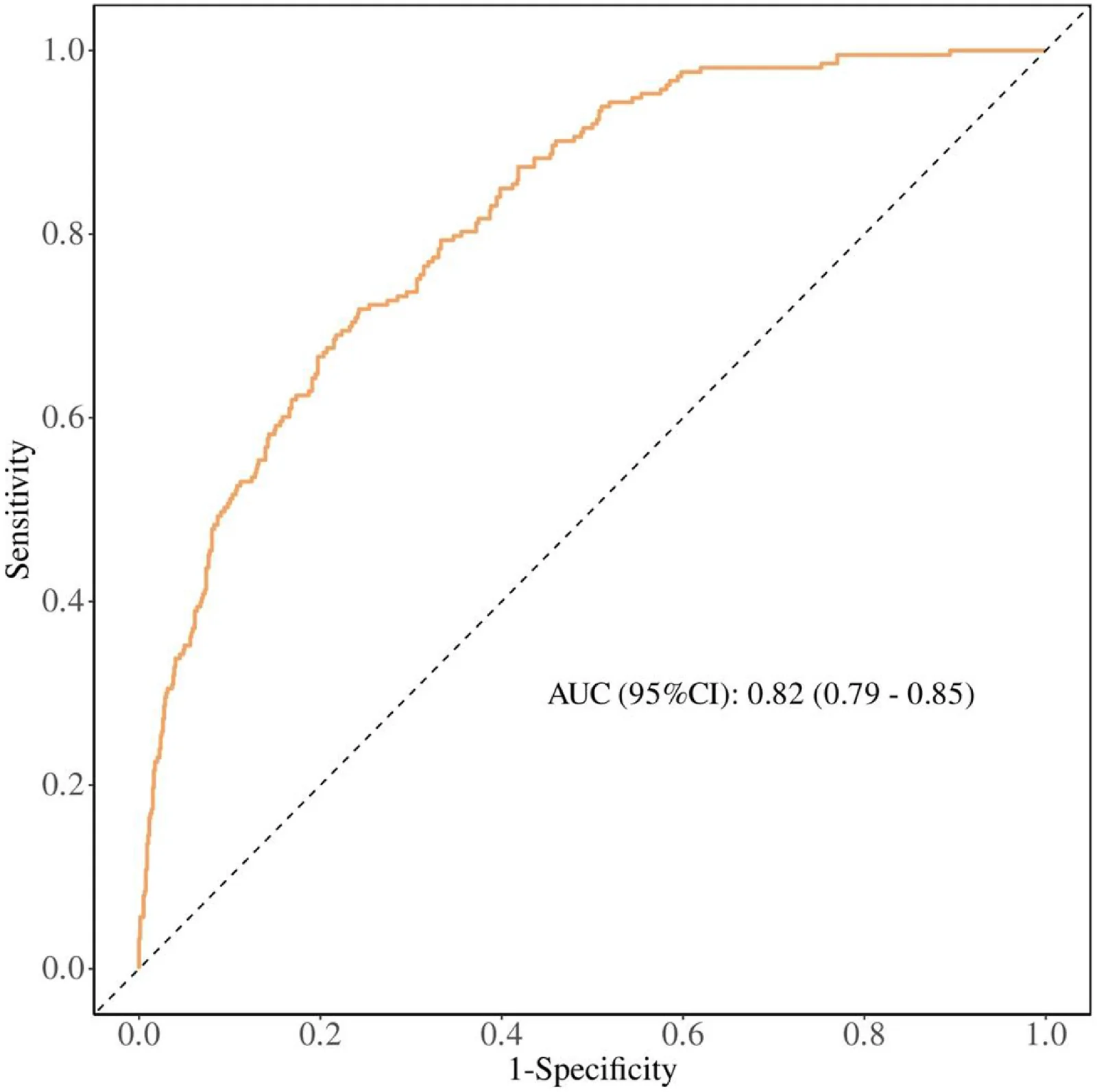
Comparison of Receiver Operating Characteristic (ROC) curves for individual serum tumor markers and the multivariable model in distinguishing lung adenocarcinoma from lung squamous cell carcinoma. The Area Under the ROC Curve (AUC) was used to quantify diagnostic performance. Comparisons between ROC curves were performed using the DeLong method.

The ROC analysis demonstrated differences across STMs in distinguishing LUAD from LUSC (Table 2). SCCA (AUC = 0.77; 95% CI: not reported) and CYFRA21–1 (AUC = 0.76; 95% CI: not reported) showed the strongest individual performance, with corresponding sensitivities of 0.82 and 0.78 and specificities of 0.79 and 0.75, respectively. NSE and ProGRP showed moderate discrimination (AUC: 0.68 and 0.60). In contrast, CEA exhibited limited discrimination (AUC = 0.55) with a low sensitivity (0.41) despite high specificity (0.80), suggesting greater utility for ruling in than for ruling out LUSC when used alone (Figs. 2, 3).

**Table 2:** Diagnostic performance of five serum tumor markers based on ROC curves.

Biomarker	AUC	Optimal Cutoff Value	Sensitivity	Specificity
CEA	0.55	1.685	0.41	0.80
CYFRA21–1	0.76	3.0	0.82	0.79
SCCA	0.77	1.0	0.78	0.75
NSE	0.68	13.5	0.66	0.64
ProGRP	0.60	43.0	0.58	0.57

ROC, Receiver Operating Characteristic; AUC, Area Under the Curve; CI, Confidence Interval; CEA, Carcinoembryonic Antigen; CYFRA21–1, Cytokeratin-19 Fragment; SCCA, Squamous Cell Carcinoma Antigen; NSE, Neuron-Specific Enolase; ProGRP, Pro-Gastrin-Releasing Peptide. Cut-off values were determined based on the Youden index.

### Univariate and multivariate models

In the univariate models, age (OR = 1.07, 95% CI 1.05–1.08, *p* < 0.001), CYFRA21–1 (OR = 1.04, 95% CI 1.02–1.05, *p* < 0.001), SCCA (OR=1.23, 95% CI 1.15–1.32, *p* < 0.001), NSE (OR = 1.02, 95% CI 1.01–1.03, *p* = 0.004), and ProGRP (OR = 1.01, 95% CI 1.01–1.02, *p* = 0.025) were significantly associated with higher odds of LUSC, whereas CEA was associated with lower odds (OR=0.97, 95% CI 0.95–0.99, *p* = 0.004) (Table 3).

**Table 3:** Univariable and multivariate logistic regression.

Variables	Single						Multivariate			
	β	S.E	Z	p	OR (95% CI)	β	S.E	Z	p	OR (95% CI)
Age	0.06	0.01	7.42	**<0.001**	1.07 (1.05∼1.08)	0.05	0.01	5.09	**<0.001**	1.05 (1.03∼1.08)
CEA	−0.03	0.01	−2.85	**0.004**	0.97 (0.95∼0.99)	−0.13	0.03	−4.10	**<0.001**	0.88 (0.83∼0.94)
CYFRA21–1	0.04	0.01	5.53	**<0.001**	1.04 (1.02∼1.05)	0.06	0.01	4.39	**<0.001**	1.06 (1.03∼1.09)
SCCA	0.21	0.04	5.68	**<0.001**	1.23 (1.15∼1.32)	0.11	0.03	3.34	**<0.001**	1.11 (1.05∼1.19)
NSE	0.02	0.01	2.87	**0.004**	1.02 (1.01∼1.03)					
ProGRP	0.01	0.00	2.24	**0.025**	1.01 (1.01∼1.02)					

OR, Odds Ratio; CI, Confidence Interval.

Bold values indicate statistical significance (p < 0.05).

In the multivariate model, age (OR = 1.05, 95% CI 1.03–1.08, *p* < 0.001), CYFRA21–1 (OR = 1.06, 95% CI 1.03–1.09, *p* < 0.001), and SCCA (OR = 1.11, 95% CI 1.05–1.19, *p* < 0.001) remained independently associated with higher odds of LUSC, whereas CEA remained inversely associated (OR = 0.88, 95% CI 0.83–0.94, *p* < 0.001). NSE and ProGRP were not statistically significant in the multivariate model (Table 3).

Logistic regression models with histology coded as LUSC = 1 and LUAD = 0. The regression coefficient (β), Standard Error (SE), Wald Z, p-value, and Odds Ratio (OR) with 95% Confidence Interval (95% CI) are shown for each predictor. The univariable results are presented for age, sex (male = 1; female = 0), CEA, CYFRA21–1, SCCA, NSE, and ProGRP. The multivariable model included age, CEA, CYFRA21–1, and SCCA.

The multivariate model included age, CEA, CYFRA21–1, and SCCA. The AUC was 0.82 (95% CI 0.79–0.85), significantly higher than any single STM (DeLong test *p* < 0.05 for all). Under the selected threshold, sensitivity was 0.76 (95% CI 0.73–0.79) and specificity was 0.72 (95% CI 0.66–0.78), indicating improved detection of LUSC while reducing the misclassification of LUAD.

Comparison of ROC curves between the multivariate model and individual serum tumor markers. The multivariate model (age, CEA, CYFRA21–1, SCCA; solid line) achieved the highest AUC 0.82 (95% CI 0.79–0.85) and outperformed each single-marker model (DeLong test *p* < 0.05).

### Calibration and clinical utility

**Fig. 4:**
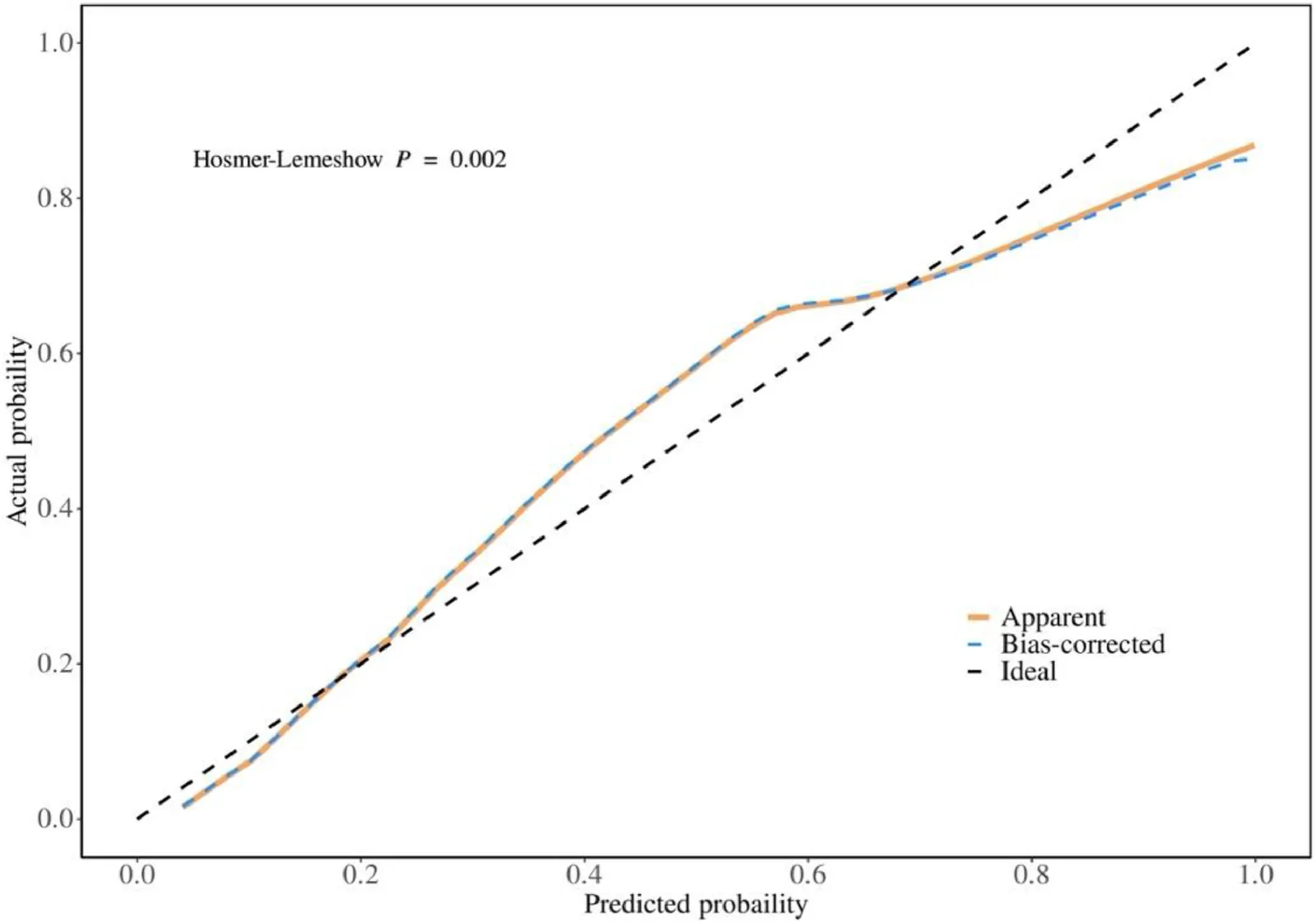
Calibration of the multivariate model. Apparent and corrected calibration curves (1000 resamples) plotted against the ideal 45° line across predicted probabilities of 0.20–0.70. Hosmer-Lemeshow p-values are shown.

The calibration curve for the multivariate model showed that the predicted probabilities fit well with the observed outcome rates. Within the probability interval of 0.2–0.7, the apparent curve and the curve after 1000 bootstrap iterations were close to the ideal 45° line, indicating good agreement between predicted and observed risks. The Hosmer-Lemeshow test yielded *p* = 0.002, indicating a minor misfit in the context of a large sample, but overall calibration was acceptable (Fig. 4).

**Fig. 5:**
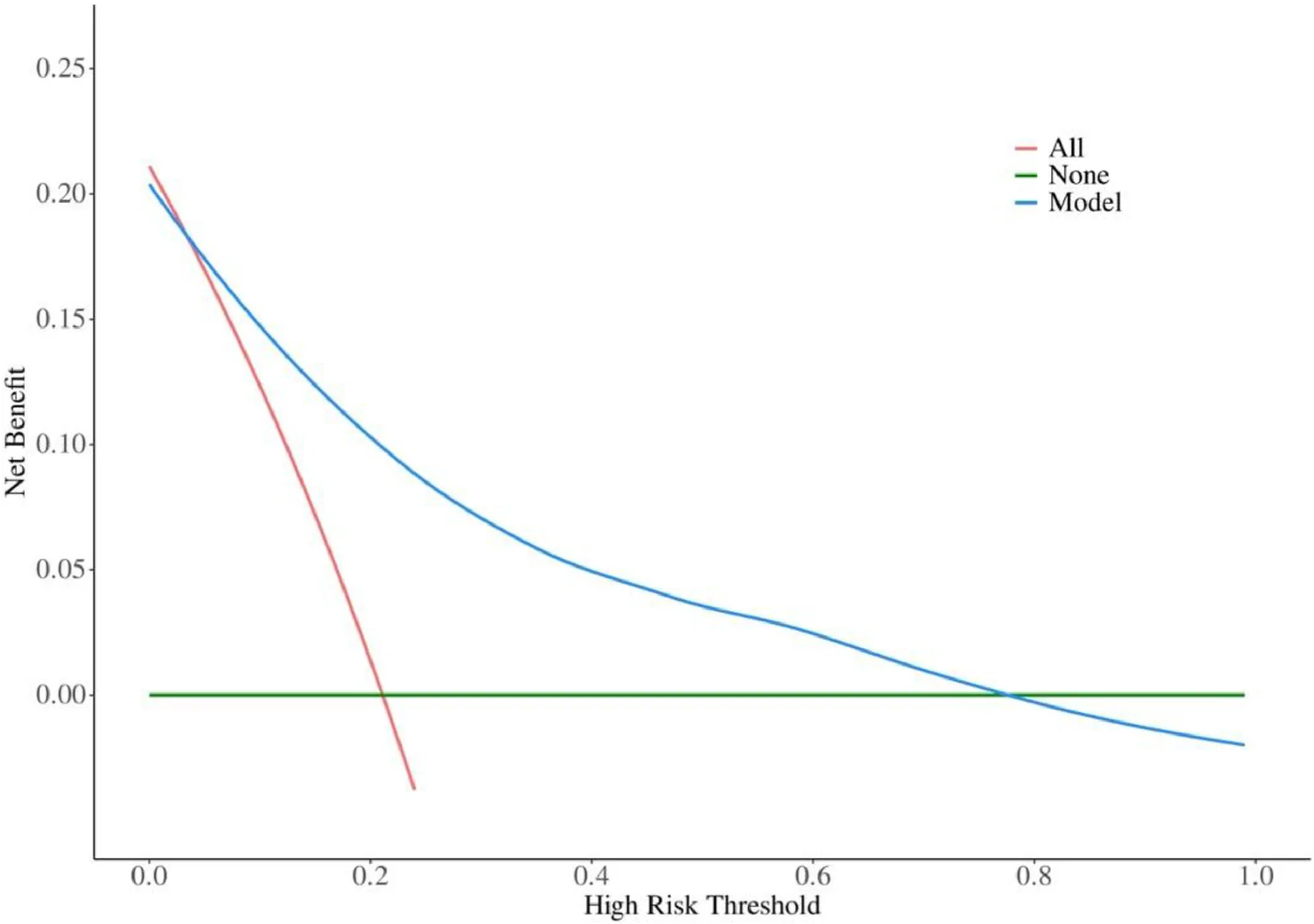
Decision curve analysis of the multivariable diagnostic model for distinguishing lung adenocarcinoma from lung squamous cell carcinoma. Net benefit was evaluated across a range of threshold probabilities and compared with strategies of performing immunohistochemistry in all patients or none.

DCA showed that, across threshold probabilities of 0.1–0.7, the multivariate model achieved higher net benefit than performing immunohistochemical work-up in all patients or none (Fig. 5). Within this range, the model can reduce the frequency and cost of unnecessary pathological examinations while enabling the identification of LUSC at clinically relevant thresholds.

Net benefit curves for the multivariate model across threshold probabilities of 0.10–0.70, compared with the benchmark approaches of performing immunohistochemical work-up in all patients or none. The serology panel was obtained first, and immunohistochemistry was performed selectively according to the predicted risk.

### Cost comparison and diagnostic workflow

In the cost comparison, the unit cost of the STM panel (CEA + CYFRA21–1 + SCCA) was approximately USD 18.6, which was lower than the cost of immunohistochemical testing (USD 140). Guided by the predictions of the multivariate model, the authors evaluated an STM-based workflow in which serological screening was performed first, followed by Immunohistochemistry (IHC). Under this workflow, projected costs were lower than those of an IHC-only approach.

## Discussion

This study systematically evaluated the baseline serum levels of CEA, CYFRA21–1, SCCA, NSE, and ProGRP in patients with NSCLC and developed a multivariate diagnostic model to differentiate between LUSC and LUAD. Rather than focusing on long-term outcomes or treatment response, the present study was designed to evaluate the clinical utility of serum tumor markers for histological subtyping at the time of initial diagnosis. The results show that the diagnostic efficacy of any single marker is limited, whereas combining multiple markers with clinical covariates can substantially improve discrimination, suggesting practical value for STM-based screening in subtyping when biopsy tissues are scarce.

In single-marker analyses, CYFRA21–1 and SCCA levels were higher in patients with LUSC, whereas CEA levels were higher in patients with LUAD, consistent with previous results.^13^, ^14^, ^15^ This difference may reflect underlying pathophysiology: glycoproteins are more actively expressed in LUAD, whereas serum markers associated with squamous differentiation ‒ particularly CYFRA21–1 and SCCA ‒ are typically higher in LUSC.^16^^,^^17^ In addition, although NSE and ProGRP are primarily associated with small cell lung cancer, they also showed between-group differences in this cohort, suggesting ancillary value for subtype discrimination in selected patients. These findings indicate that individual serum tumor markers provide limited but complementary biological information for NSCLC subtyping.

The multivariate model achieved higher overall discrimination (AUC=0.82) and greater net benefit than any single STM. This improvement highlights the advantage of integrating multiple biomarkers with clinical variables, rather than relying on single-marker testing alone. This finding is consistent with studies assessing the diagnostic value of multiple STMs,^18^, ^19^, ^20^, ^21^ indicating that integrating complementary markers can effectively compensate for the shortcomings of a single test and improve the discrimination between LUSC and LUAD.

In clinical practice, accurate histological classification is important for treatment decisions. Patients with LUAD often have driver gene mutations that can benefit from targeted therapy, whereas LUSC is more commonly treated with chemotherapy or immunotherapy.^2^^,^^22^^,^^23^ Therefore, in cases where pathological samples are insufficient or the diagnosis is uncertain, a combination of STMs can provide additional diagnostic information, particularly when access to tissue samples is limited and obtaining samples is difficult or unsafe. In this context, STM-based models should be considered complementary tools to support pathological assessment, rather than substitutes for histological diagnosis.

In this study, the authors evaluated the discriminative power of a multivariate model and assessed its clinical applicability through calibration curves and DCA. Calibration analysis showed that the predicted probabilities were consistent with the observed outcome rates across most probability intervals, suggesting that the predicted probability has clinical utility. Although the Hosmer-Lemeshow test suggests a slight misfit, it is sensitive to small deviations in large samples. Accordingly, graphical calibration and overall model performance should be interpreted alongside formal statistical testing, rather than relying solely on a single p-value.

DCA demonstrated clinical utility. Across thresholds of 0.1–0.7, the multivariate model yielded a higher net benefit than performing immunohistochemical work-up in all patients or none. Within this range, using the model to guide serological screening can potentially reduce the frequency and cost of unnecessary pathological examinations while enabling the diagnosis of LUSC, supporting informed diagnostic and treatment decisions. These results emphasize the potential role of the model in clinical decision-making by balancing diagnostic benefit against unnecessary testing.

The cost comparison and the simulation of an STM-based workflow highlight potential gains in resource utilization. Compared with an IHC-only approach, STM-based screening followed by IHC reduced costs and may decrease the number of invasive procedures. These findings are particularly relevant in resource-limited settings. From a health economics perspective, this stepwise diagnostic strategy may help optimize the allocation of pathological resources in routine practice.

This study has limitations. First, the single-center retrospective design may introduce selection and spectrum bias; therefore, multicenter, large-sample prospective studies in clinical settings are warranted to validate the findings. In addition, the relatively short study period precluded evaluation of long-term outcomes such as treatment response or disease recurrence. Second, only conventional STMs were assessed; emerging molecular markers (circulating tumor DNA and exosome markers) could be integrated to enhance discrimination.^24^, ^25^, ^26^ Third, the present diagnostic model requires external validation in independent cohorts to ensure generalizability; performance may require recalibration and workflow adjustments according to disease prevalence, testing costs, and clinical practice patterns across different populations.

## Conclusion

Although the serum levels of CEA, CYFRA21–1, and SCCA vary by histological subtype, the performance of these markers in distinguishing between LUSC and LUAD was limited. In contrast, a diagnostic model containing multiple markers can improve diagnostic accuracy, sensitivity, and specificity. This model can be used as an adjunct when tissue specimens are insufficient or unobtainable. Multicenter, large-sample prospective studies using an STM panel combined with new molecular markers are needed to enhance the clinical applicability of this diagnostic model.

## Data availability

The data supporting the findings of this study are available within the article.

## Authors’ contributions

Zhuoying Chen: Conceptualization; methodology; data curation; formal analysis; writing-original draft; visualization. Li Chen: Data curation; investigation; resources; validation. Lichun Wu: Investigation; resources; data curation. Ke Zhang: Conceptualization; supervision; project administration; writing-review & editing.

## Declaration of competing interest

The authors declare no conflicts of interest.

## References

[1] Bray F., Laversanne M., Sung H. (2024). Global cancer statistics 2022: GLOBOCAN estimates of incidence and mortality worldwide for 36 cancers in 185 countries. CA Cancer J Clin.

[2] Huang Q., Li Y., Huang Y. (2025). Advances in molecular pathology and therapy of non-small cell lung cancer. Signal Transduct Target Ther.

[3] Gálffy G., Morócz É, Korompay R. (2024). Targeted therapeutic options in early and metastatic NSCLC-overview. Pathol Oncol Res.

[4] Lau S C M, Pan Y., Velcheti V. (2022). Squamous cell lung cancer: current landscape and future therapeutic options. Cancer Cell.

[5] Ngwa V M, Hwang Y., Song W. (2025). Targeting mTORC2 in lung squamous cell carcinoma improves anti-tumor immunity through the PSGL-1-VISTA axis. Cancer Gene Ther.

[6] Kim S H, Kim M H, Lee M K (2023). Problems in the pathologic diagnosis of suspected lung cancer. Tuberc Respir Dis.

[7] Dou X., Lu J., Yu Y. (2024). Determination of tumor marker screening for lung cancer using ROC curves. Dis Markers.

[8] Zhou Y., Tao L., Qiu J. (2024). Tumor biomarkers for diagnosis, prognosis and targeted therapy. Signal Transduct Target Ther.

[9] Bi H., Yin L., Fang W. (2023). Association of CEA, NSE, CYFRA 21-1, SCC-Ag, and ProGRP with clinicopathological characteristics and chemotherapeutic outcomes of lung cancer. Lab Med.

[10] Lv H., Luo P., Wang J. (2025). Diagnostic efficiency of peripheral tumor serum biomarkers in lung cancer and their correlation with clinicopathological features. Am J Transl Res.

[11] Wu L., Chen X., Peng T. (2024). Human epididymal protein 4 and its combined detection show good diagnostic value in lung cancer: a retrospective study. Int J Biol Markers.

[12] Hu S., Guo Q., Ye J. (2024). Development and validation of a tumor marker-based model for the prediction of lung cancer: an analysis of a multicenter retrospective study in Shanghai. China. Front Oncol..

[13] Kim Y C, Park K O, Choi I S (1996). A comparison of serum CYFRA 21-1 and SCC Ag in the diagnosis of squamous cell lung carcinoma. Korean J Intern Med.

[14] Kataoka N., Katayama Y., Yamada T. (2024). CYFRA 21-1 predicts efficacy of combined chemoimmunotherapy in patients with advanced non-small cell lung cancer: a prospective observational study. Transl Lung Cancer Res.

[15] Sun H., Wu S., Chen Z. (2025). Predictive value of perioperative carcinoembryonic antigen changes for recurrence in non-small cell lung cancer. Transl Lung Cancer Res.

[16] Frohwitter G., Buerger H., PJ V A N D (2016). Cytokeratin and protein expression patterns in squamous cell carcinoma of the oral cavity provide evidence for two distinct pathogenetic pathways. Oncol Lett.

[17] Stowell S.R., Ju T., Cummings R D (2015). Protein glycosylation in cancer. Annu Rev Pathol.

[18] Wang X., Zhi X., Yang Z. (2017). A novel serum-based biomarker panel has complementary ability to preclude presence of early lung cancer for low dose CT (LDCT). Oncotarget.

[19] Yao L., Li Y., Wang Q. (2023). Multi-biomarkers panel in identifying benign and malignant lung diseases and pathological types of lung cancer. J Cancer.

[20] Izawa A., Hara Y., Horita N. (2024). Improved diagnostic accuracy with three lung tumor markers compared to six-marker panel. Transl Lung Cancer Res.

[21] Sua L F, Serrano-Gomez S J, Nuñez M. (2024). Diagnostic potential of protein serum biomarkers for distinguishing small and non-small cell lung cancer in patients with suspicious lung lesions. Biomarkers.

[22] Bai Y., Yang W., Käsmann L. (2024). Immunotherapy for advanced non-small cell lung cancer with negative programmed death-ligand 1 expression: a literature review. Transl Lung Cancer Res.

[23] Su P L, Furuya N., Asrar A. (2025). Recent advances in therapeutic strategies for non-small cell lung cancer. J Hematol Oncol.

[24] Ying L., Du L., Zou R. (2020). Development of a serum miRNA panel for detection of early-stage non-small cell lung cancer. Proc Natl Acad Sci U S A..

[25] Lam W K J, Bai J., Ma M L (2024). Circulating tumour DNA analysis for early detection of lung cancer: a systematic review. Ann Transl Med.

[26] Thuya W L, Peyper J M, Myen T T (2024). Exosome autoantibody biomarkers for detection of lung cancer. Mil Med Res.

